# Next-Generation swimming pool drowning prevention strategy integrating AI and IoT technologies

**DOI:** 10.1016/j.heliyon.2024.e35484

**Published:** 2024-07-31

**Authors:** Wei-Chun Kao, Yi-Ling Fan, Fang-Rong Hsu, Chien-Yu Shen, Lun-De Liao

**Affiliations:** a Institute of Biomedical Engineering and Nanomedicine, National Health Research Institutes, 35 Keyan Road, Zhunan Town, Miaoli 350, Taiwan; b Department of Information Engineering and Computer Science, Feng Chia University, Taichung 407, Taiwan; c Department of Biomedical Engineering & Environmental Sciences, National Tsing-Hua University, Hsinchu, Taiwan

**Keywords:** Drowning prevention, Preventive measures, Safe practice, Water emergency, Internet of things, Artificial intelligence

## Abstract

Drowning, as a leading cause of unintentional injury-related deaths worldwide, is a major public health concern. Swimming pool drowning is the main cause of most drowning incidents, and even with preventive measures such as surveillance cameras and lifeguards, tens of thousands of lives are lost to drowning every year. To address this issue, technology is being utilized to prevent drowning accidents and provide timely alerts for rescue. This paper explores the use of drowning prevention technology in embedded systems within enclosed environments, artificial intelligence (AI), and the Internet of Things (IoT) to decrease the likelihood of drowning incidents. Embedded systems play a critical role in such technology, enabling real-time monitoring, identification of dangerous situations, and prompt alerting. Due to their ease of installation and technical implementation, embedded devices are especially effective as drowning prevention devices. The image recognition capabilities of drowning prevention systems are enhanced through computer vision. Swimming pool drowning situations can be identified with the help of cameras and deep learning technologies, thereby increasing rescue efficiency. Finally, the IoT endows drowning prevention systems with comprehensive intelligence by connecting various devices and communication tools. Real-time alert transmission and analysis have become possible, enabling the early prediction of dangerous situations and the implementation of preventive measures, significantly reducing drowning incidents. In summary, the integration of these three types of drowning prevention technologies represents significant progress. The flexibility, accuracy, and intelligence of drowning prevention systems are enhanced through the incorporation of these technologies, providing robust support for safeguarding human lives and thus potentially saving tens of thousands of lives each year.

## 1 Introduction

### 1.1 Research on the prevention of drowning incidents

**Fig. 1:**
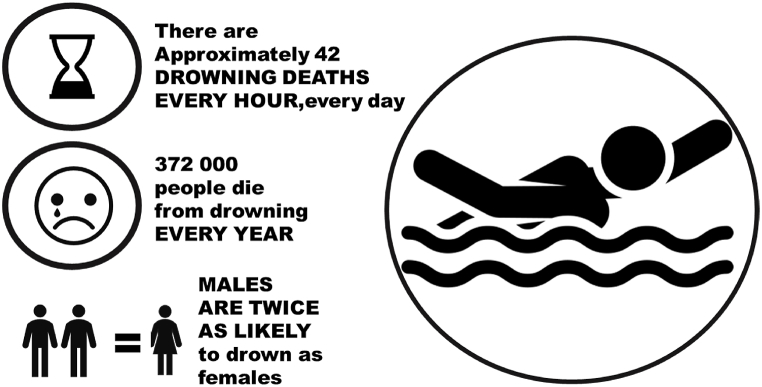
Drowning incidents are fast-paced events, and people can be exposed to unsafe conditions at any time. Because most people have not received complete drowning prevention training, it is often difficult to recognize what drowning looks like. This is particularly significant because when discovering a drowning individual, it is often difficult to immediately identify the specific situation they are experiencing. This difficulty can lead to significant delays in initiating rescue actions, thereby drastically reducing the golden time for rescuing them from drowning. Drowning is a significant global issue that demands attention. Statistical data reveal that approximately 42 drowning deaths occur every hour, resulting in approximately 37,200 deaths per year worldwide. Moreover, it is alarming to note that the incidence of drowning is twice as high in males than in females, making it a major global public health concern. The urgency of addressing these incidents becomes apparent when considering the importance of prompt and effective rescue efforts for mitigating the severity of situations and saving lives [5]. To address this global public health issue, concerted actions are necessary. This involves providing broader safety education, enhancing people's swimming skills, improving surveillance and rescue facilities in water environments, and increasing public awareness regarding the dangers of drowning. Implementing comprehensive strategies that encompass education, skill building, and infrastructure improvements is crucial for reducing the incidence of drowning and improving overall water safety. This multifaceted approach can contribute to creating a safer environment, fostering a culture of water safety, and ultimately saving lives.

Drowning is a significant global public health issue; approximately 236,000 people lost their lives to drowning in 2019 alone, as reported by the United Nations Global Drowning Report [1,2]. According to the Global Burden of Disease (GBD) 2019 estimates, drowning ranks as the second leading cause of unintentional injury-related deaths after falls, adjusted for age-standardized rates per 100,000 people [3]. In 2019, over 230,000 people worldwide died from drowning, with the majority of deaths occurring in middle-income or low-income countries, accounting for 7 % of all injury-related deaths [4]. As shown in Fig. 1, drowning is the third leading cause of accidental injury-related deaths globally [5,6]. In 2000, according to the Global Burden of Disease 2000 (GBD 2000) data, approximately 449,000 people worldwide lost their lives due to drowning. This represents a rate of 7.4 deaths per 100,000 people. Furthermore, a staggering 1.3 million disability-adjusted life years (DALY) were lost as a result of premature deaths or disabilities caused by drowning. Notably, an overwhelming majority of these tragic incidents, precisely 97 %, occurred in low- and middle-income countries [7]. Accidental drowning incidents are significant causes of death, premature mortality, and disability among adolescents in the Americas [8]. The prevention of swimming pool drownings is a global issue that requires comprehensive solutions. World Health Organization (WHO) statistics show that the mortality rate due to drowning is highest among children under 5 years old, regardless of their gender. Even in shallow or infant pools, toddlers can drown silently in just 25 s. Among children under 15 years old, drowning has a higher mortality rate than any other cause of injury-related death. In Los Angeles, almost half of all drowning incidents occur in swimming pools. Drowning is now the second-leading cause of unintentional death in children and the second-leading cause of potential loss of life. Among all causes of child mortality, only car accidents and cancer surpass drowning [9]. In terms of drowning prevention awareness, a survey conducted among adult beachgoers in New Zealand (sample size: 3371) assessed beach swimming frequency, swimming skills, swimming behaviors, and perception of drowning risk across five pre-validated scenarios. The results revealed that 32 % of beachgoers estimated that they were unable to swim beyond 25 m, 26 % of people engaged in swimming after consuming alcohol, and a significant 55 % of people swam in areas not supervised by lifeguards [10]. Brenner et al. suggested that “enhancing swimming proficiency should be a vital component of aquatic competence, but it should be recognized that relying solely on swimming ability is insufficient to prevent swimming drowning” [11].

Drowning incidents occur rapidly and quietly, unlike the expected scenarios for most people. In the early stages, drowning is challenging to identify, and lifeguards may easily overlook it. This presents a significant risk, as lifeguards or nearby swimmers may notice that something is wrong, but discerning whether the issue is a drowning incident can be difficult. The uncertainty surrounding drowning makes a swift rescue operation crucial. Therefore, research must shift from detecting drowning incidents to preventing them before they occur.

Generally, people engaged in swimming activities may face the danger of drowning within a few minutes. The probability of a successful rescue and postrescue survival largely depends on the speed at which assistance reaches the victim and the efficiency of resuscitation. Some prevention strategies, such as installing barriers in swimming pools to prevent drowning incidents, have seen success. However, these successes have mostly been observed in high-income countries. In lower- and middle-income countries, financial constraints often limit the ability to significantly invest in such improvements. Instead, these countries tend to focus on public health campaigns to reduce drowning-related mortality rates [12]. Drowning while swimming is a serious concern, and certain factors increase the risk of drowning. To prevent drownings in swimming scenarios, it is important to address these risks. In countries with higher income levels, various strategies have been implemented to reduce the probability of drowning incidents. For example, barriers have been set up to restrict access to open water, effectively minimizing the risk of exposure to drowning [13]. Education is used to enhance the swimming skills of swimmers, with the aim of reducing the likelihood of drowning incidents [12]. However, drowning incidents can be caused by various factors, not just physical or cognitive limitations. They can occur unexpectedly and in any location. As a result, it is crucial to implement a comprehensive and integrated drowning prevention and detection system.

### 1.2 Applications of artificial intelligence in various fields

In the past, before the 21st century, artificial intelligence (AI) technologies were not fully developed, and computers lacked significant computational power and parallel processing speed. As a result, the safety measures employed in drowning prevention systems at swimming facilities had major limitations. During that time, the main focus was on improving hardware facilities and raising awareness about safety to protect swimmers [[14], [15], [16]]. However, with the arrival of the 21st century, remarkable advancements have been achieved in terms of integrating AI technologies across different fields such as healthcare [17], financial transactions, and transportation. AI is not only used in traditional machine learning [[18], [19], [20]] or deep learning techniques [21]. The technology is also included in natural language processing, computer vision and other fields. In 2019, drowning while swimming also became a globally recognized and critical issue. Countries worldwide have dedicated efforts to leveraging AI in conjunction with drowning prevention systems to reduce the probability of drowning incidents. Drowning incidents do not follow a fixed pattern, and human eyes can make identification errors, significantly shortening the time in which a person must be rescued when drowning occurs. It is imperative to integrate artificial intelligence and algorithmic technologies into drowning prevention systems, utilizing high-speed computations and real-time alerts to notify staff, thereby further safeguarding the lives of swimmers. AI has become an indispensable part of our lives, and its rapid development is transforming our daily experiences. This technology brings unprecedented convenience and enhances our ability to protect our safety.

AI is a broad term that can be interpreted as the use of computer programming to train machines and execute tasks. It involves the development of algorithms and techniques that enable machines to engage in activities such as inference, cognitive reasoning, and conscious reasoning [22]. The field is witnessing significant advancements that are shaping the development of AI technologies. These innovations are gradually replacing and enhancing human abilities in various fields, bringing about a profound impact on our everyday lives. From areas such as big data to medical imaging and engineering [23], AI has proven to be highly efficient, providing predictive capabilities that greatly contribute to convenience and productivity [24].

Big data can also be applied in the medical field [25], and this information can be used to establish psychological counseling platforms or medical advisory services [26]. By leveraging big data and AI technology, comprehensive health information can be collected to allocate prioritized medical resources [27,28], assist in classifying medical images [29] and provide the computing services needed for traditional medicine [30]. This includes the widespread application of wearable devices in healthcare [31,32] and medical applications combined with the Internet of Things (IoT) [33,34]. An artificial intelligence of things (AIoT) system has been utilized for electrocardiograph (ECG) analysis [35] and cardiac disease detection. This system comprises IoT-based front-end hardware, a user interface on a communication device application (APP), a cloud database, and an AI platform for cardiac disease detection. The IoT-based front-end hardware consists of a wearable ECG patch, which includes analog front-end circuits and a Bluetooth module that is capable of detecting ECG signals. The associated smart device application not only displays the user's real-time ECG signals but also provides real-time annotations for abnormal signals, enabling instant disease detection [36].

**Fig. 2:**
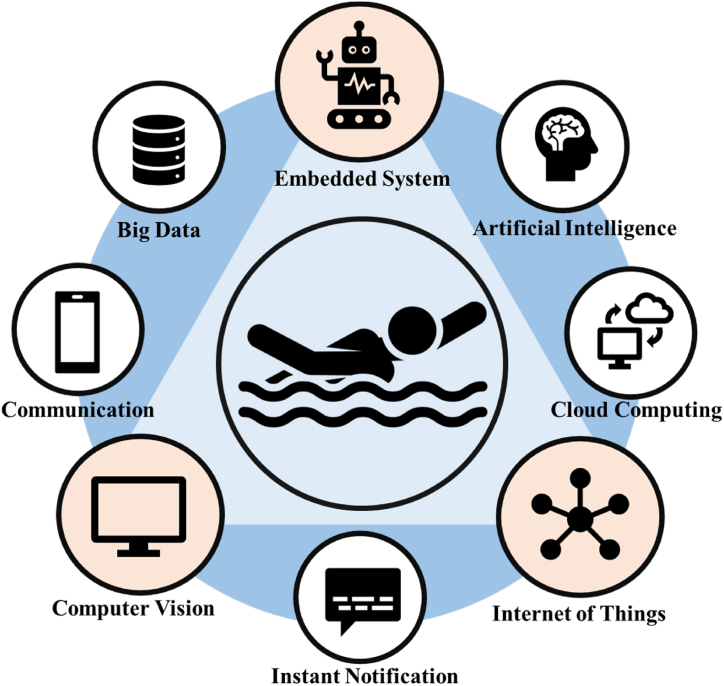
Drowning prevention is used in eight fields. Embedded Systems: Utilizing embedded systems, small sensors can be worn by swimmers to monitor their physiological data in real time. AI: AI enables simultaneous monitoring, prediction, and alarm response, significantly reducing the probability of drowning incidents. Cloud Computing: When addressing massive datasets, cloud computing is employed to conduct extensive data analyses, enhancing the accuracy of data analytics. IoT: The IoT connects a system to household devices or smartphones, allowing family members to observe the movement statuses of their loved ones at any time. Real-Time Notifications: Such a system can integrate with applications to send urgent messages to family members during drowning incidents, facilitating prompt rescue efforts. Computer Vision: The strategy of using images for recognition adapts well to diverse environments, improving the adaptability of the constructed system to different settings. Communication: The current 5G communication technology, which is 10 times faster than 4G communication, facilitates immediate communication to alert personnel when a drowning incident occurs, enabling a swift rescue. Big Data Analysis: Analyzing a large amount of drowning data helps identify behavior patterns, aiding in predicting future events and enacting preventive measures. Currently, embedded systems, the IoT, and computer vision are the main focuses, with computer vision applications being the most widely utilized approaches.

Recently, artificial intelligence technology has matured and come to be widely applied, as shown in Fig. 2. Integrating AI into drowning prevention systems enables real-time learning regarding swimming environments in different settings. This significantly reduces the probability of drowning incidents. An AI-based drowning detection system can achieve fast, accurate, and real-time drowning detection. Combining AI with drowning prevention systems addresses the lack of real-time alerts and enables these systems to adapt to various swimming environments, greatly enhancing their adaptability to different scenarios. This research aims to address the prevalence of drowning by utilizing AI for drowning prevention. By integrating the advantages of big data technology with the current limitations of drowning prevention products, the goal is to seamlessly blend technology and design [37].

However, public areas such as swimming pools can become very crowded during holidays or when the weather is hot. Insufficient manpower may prevent the complete surveillance of activities in aquatic facilities, preventing people from engaging in swimming activities in a safe environment. The lack of public knowledge about drowning and the inability to recognize drowning situations further compound the problem. Therefore, the development of precise sports science-based drowning prevention technologies is crucial for the future. By utilizing precise sports science to observe the real-time activity-related data of people, the chances of successfully rescuing a drowning person can be increased. This approach aims to reduce the demand for lifeguard manpower by assisting lifeguards in monitoring the activity statuses of people in pool areas. The application of precise sports science in drowning prevention technologies has the potential to significantly decrease the needed amount of manpower and reduce the occurrence of drowning incidents. The application of drowning prevention technologies based on modern smart technologies, including embedded systems, computer vision, and the IoT, will be explored in this paper, highlighting their contributions and potential for designing effective drowning prevention systems.

## 2 Preventing drowning through embedded system-based approaches utilizing development boards and sensors to transmit signals

**Fig. 3:**
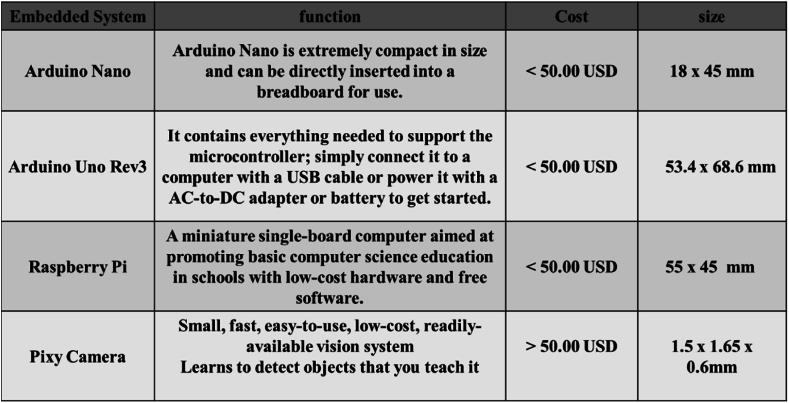
The development and progress achieved by embedded systems have brought numerous benefits to various industries. Today, we have easy access to a range of embedded systems, such as Arduino Nano, Arduino Uno, Raspberry Pi, and Pixy Camera. Each system has its own advantages in different areas. From a cost standpoint, these embedded systems are generally affordable, allowing more people to engage in the computer vision and IoT fields. This reduced cost barrier promotes innovation and experimentation.

Drowning claims a significant number of lives each year. In the past, efforts to prevent and detect drownings primarily centered around optimization ideas in embedded systems. These optimization processes involved utilizing sensors, such as ultrasonic sensors, along with development boards and other technologies to achieve improved drowning prevention and detection (as shown in Fig. 3).

For instance, this type of system utilizes components such as a Raspberry Pi with the Raspbian operating system, a Pixy camera, an Arduino Nano board, a stepper a motor, an alarm system, and a motor driver. Such a system performs real-time calculations using color blocks to locate and rescue drowning swimmers while simultaneously alerting the staff [38]. Radar is widely used for human activity recognition due to its powerful ability to capture micro-Doppler features and its strong environmental adaptability. In one work, a novel radar-based potential drowning detection system was proposed. To achieve enhanced cross-domain fusion efficiency and intradomain feature learning, a two-stage fusion network was designed for the drowning detection system [39].

In sensor applications, methods combining image processing, accelerometers, pulse and pressure sensors, and LASER light-dependent resistor (LDR) technology have been utilized [40]. Due to the higher risk of drowning in infants than in adults, a wearable wristwatch was employed to record variations in a swimmer's blood pressure and heartbeat. By using an Arduino Uno microcontroller, the received signals were processed, and a warning signal was triggered through a buzzer [41]. The advantage of wearable devices is their noninvasive nature, preventing discomfort for swimmers while enhancing their safety during activities. When a swimmer is drowning, a safety device on their wrist can inflate safety airbags, assisting the drowning individual in maintaining a floating position [42]. Wearable Bluetooth tags periodically send beacon signals to a Bluetooth beacon scanner. In one study, an improved RSSI-based algorithm was employed to estimate the position of a swimmer. When a victim remains submerged underwater for a certain duration, the wireless link between the Bluetooth tag and the beacon scanner is disrupted. Subsequently, alarms placed in the monitoring area are triggered [43]. Upon detecting a swimmer's distress based on body movements, the sensors trigger the automatic deployment of the safety airbag in the device when the system identifies a drowning situation. This principle is similar to the mechanism used by airbags in car driver seats [44]. Moreover, in addition to devices worn on the wrist, researchers are also investigating the concept of placing a device on the user's chest. Such a device utilizes pressure sensors, accelerometers, and gyroscopes to assess a swimmer's behavior and determine their risk of drowning [45]. Setups that involve placing ultrasonic sensors in a swimming pool have also been used to determine the swimmer's state based on a threshold. Sensor technology can be employed to differentiate whether a drowning object is a person or another object [46]. J. Misiurewicz developed a drowning detection system by creating a model that responds to a swimming pool environment. The distinctive feature of a swimming pool environment is the presence of large and flat reflective surfaces surrounding a small amount of water. However, the reflection from the water surface in a swimming pool is larger and clearer than those in typical water sound applications [47].

Embedded systems can incorporate hardware sensors within a venue to provide enhanced safety measures. For example, ultrasonic sensors can be utilized to detect drowning instances. When a sensor identifies a potential drowning incident, an alarm is activated, and a device such as a tablet is deployed to assist in retrieving the person from the water and prevent drowning. This type of system aims provide improved real-time responses to drowning incidents and offer effective rescue solutions [48]. Ultrasonic sensors utilize receivers and transmitters to determine the positions of swimmers. Through the use of acoustic simulation, the system can analyze the distance information contained in ultrasonic waves. This process helps verify the ability of an algorithm to accurately calculate the 3D positions of swimmers [49]. Currently, sensors can also capture physiological data. By utilizing pulse oximetry technology, sensors can measure a participant's heart rate and the oxygen saturation level in their blood in real time. This allows for monitoring the swimmer's physiological state [50]. Furthermore, a sensor-based swimming state classification and movement distance estimation method was proposed. Drawing inspiration from the concept of pedestrian dead reckoning (PDR), an algorithm for swimmer localization was introduced in another study [51].

Swimming pools can now benefit from cutting-edge safety systems that are fully automated. This advanced technology continuously monitors the oxygen levels of swimmers. If these levels exceed a predetermined threshold, an automatic safety net is activated to provide additional security. Furthermore, when a pool is not being used, the safety net is deployed to effectively cover and protect it [52]. Furthermore, in terms of swimming posture, the classification task can be based on three different swimming styles: the freestyle, butterfly, and backstroke styles. Experimental studies conducted in swimming pools have captured the acoustic Doppler features generated by different swimming styles. A spectrogram can be treated as an image, and in such cases, applying a dynamic convolutional neural network (DCNN) [53] proves effective for performing feature recognition [54]. A camera with an integrated wristband can be employed to recognize the drowning postures and pulse signals of the human body. This method features accurate determination, fast execution, and convenient self-rescue capabilities in rescue operations [55].

Furthermore, by installing embedded systems underwater and utilizing underwater sonar for scanning purposes, accurate drowning detection can be achieved through deep neural networks. One such system achieved 88 % classification accuracy in a scanning time of 1.5 s [56]. This implementation method is relatively unique compared to the aforementioned approaches, and the use of underwater sonar for scanning is a relatively uncommon approach, warranting further research.

## 3 Computer vision-based deep learning applications for drowning image recognition

In recent years, the widespread and extensive application of AI has led to advancements in many traditional detection technologies. The integration of artificial intelligence technologies has provided increased computing speeds. In image segmentation [57], object detection technology is the foundation of the artificial intelligence field [58]. In computer vision, a widely utilized model is You Only Look Once (YOLO). The first YOLO model was introduced in a paper published by Joseph Redmon and others in 2015 [59]. YOLO is a novel object detection method. The foundational YOLO model processes images at a speed of 45 frames per second (FPS). Taking the YOLO model as an example, a new drowning risk detection method was proposed using YOLOv4 [60] and a MA_CBAM module. This approach aims to construct an effective drowning warning system. Experimental results demonstrated that the improved model, MA_CBAM-YOLOv4, performs well in comparison with the original YOLOv4 method, exhibiting higher accuracy and robustness [61]. In real-life scenarios, the probability of drowning is higher for infants than adults. To achieve rapid drowning detection for infants in the real world, a study explored the advantages of the faster region-based CNN (Faster R–CNN) [62] and a series of YOLOv5 models. In the YOLOv5 series, both the Faster R–CNN and YOLOv5s achieved mean average precision (mAP) values exceeding 89 %. The former processed only 6 FPS with an accuracy of 62.04 %, while the latter achieved an average speed of 75 FPS with an accuracy rate of approximately 86.6 % [63]. Additionally, a swimmer behavior recognition framework was developed based on the YOLOv4 algorithm (BR-YOLOv4). The model analyzes the location information of the target and the relationship between the swimming area and the drowning area. Different detection methods have also been evaluated in terms of accuracy [64]. By utilizing the 93.48 % area under the curve (AUC) of YOLOv2 [65], swimmers can be accurately detected under very challenging conditions. Tiny-YOLO obtained a 79.29 % AUC, and it can be implemented on low-cost embedded systems, enabling them to generate on-site results in real time [66].

The mask region-based CNN (Mask R–CNN) has also been utilized [67]. By incorporating features into a pyramid model to optimize the main convolutional architecture of the traditional Mask R–CNN algorithm, a drowning detection system for swimmers was designed. Experimental results indicated that the system achieved a detection speed of 6 FPS, a detection rate of 94.1 %, and a false detection rate of 5.9 % [68]. Recently, image recognition technology has gradually matured, and it can adapt to various environments or pictures in various fields. By integrating the computer vision aspects of camera technology, for example, a method for detecting drowning in images was designed. The constructed dataset included three main water activity behaviors (swimming, drowning, and safe) captured by overhead and underwater cameras, and two drowning detection methods were developed and tested on the proposed dataset [69]. Underwater cameras can also be combined with OpenPose [70] features when the cameras are positioned at higher angles, and it becomes challenging to accurately capture a swimmer's entire posture, ultimately reducing the accuracy of the system. To overcome this challenge, the system employs image joint point features to aid in recognizing the swimmer. This approach ensures that all of the swimmer's joint point movements are completely captured. The captured joint point features are then input into a recurrent neural network (RNN) to determine if the swimmer is drowning. Based on its training results, the system achieved an accuracy rate of approximately 89.4 % [71].

A computer vision system based on camera lenses mainly consists of visual components and an event inference module. The event inference module can analyze observed sequences of swimming features, while the visual components can distinguish between the background pool area and the foreground swimmer based on a model [72,73]. The hidden Markov model (HMM) was utilized to identify signs of drowning to analyze the motion behaviors of swimmers [74]. The camera can also monitor the positions of people in the water and report potential drowning events. This system can be implemented through computer vision and neural networks, creating a neural network model with real-time processing that is capable of handling multiple consecutive frames [75]. In harsh environments, an automated monitoring system is needed to detect water crises in highly dynamic aquatic settings. One system was developed based on robust background modules and efficient segmentation methods, ensuring reliable user detection in reflections, ripples, splashes, and rapid lighting changes. This system can produce results in challenging detection environments [76].

A novel technology built on camera-based drowning detection algorithms was also developed. The input of this algorithm consists of footage captured by underwater cameras, and moving objects in the alert zone of the pool are extracted from the background using background subtraction [77]. F. Lei et al. applied a Kalman filter to underwater surveillance cameras. Through experiments conducted on multiple underwater videos, the model was found to effectively adapt to underwater environments [78]. A drowning detection method based on background subtraction was also proposed, where each pixel is described using a Gaussian mixture model. An adaptive background model is established, and it is updated in real time [79]. To extract the background and use the frame difference vibration algorithm to update the precise motion region throughout the entire video, this method was employed to detect static and dynamic features for identifying normal swimmers and drowning people. This approach was used to improve upon the results of the traditional Visual Background Extractor (ViBe) algorithm, where the camera was installed above the water surface in real time to capture image sequences of a pool and then swimmer detection, swimmer tracking, and drowning analysis were performed [80]. The drowning detection device, composed of a camera integrated with embedded AI devices and a waterproof housing, utilizes underwater computer vision for drowning detection. By means of the exceptional high-performance computing capabilities of the Jetson Nano, this cutting-edge detection device is able to process and analyze complex data at remarkable speeds. Its advanced computing abilities are made possible by its powerful GPU architecture, which allows it to perform calculations with unparalleled accuracy, even when dealing with large datasets. As a result, the detection device is able to produce precise and detailed results in a fraction of the time it would take traditional devices to complete the same task. To address environmental interference around the pool, a two-stage approach is employed. In the first stage, the YOLOv5n network is used to detect nearly vertical human bodies. In the second stage, a lightweight drowning detection network called DDN (a DCNN) based on a deep Gaussian model is used for fast feature vector detection [81]. Moreover, in unmanned recreational areas, the implementation of a novel pool image monitoring system for automatic drowning event detection can effectively integrate a background model. This type of system remains reliable in terms of detecting and tracking swimmers, even in the presence of visual noise such as water ripples, splashes, and shadows [82]. Additionally, drones can be integrated with CNN [83,84] models to detect swimmers. This integration process involves three stages [85]. The system utilizes airborne sliding gesture detection to assist users in making gestures in front of the camera and executing different actions. OpenCV software is used to detect changes in the environment [86].

In terms of swimming images, segmentation is performed on early-stage drowning images obtained in a swimming pool. This segmentation technique is based on automatically generating a pool mask to separate the aquatic area from the remainder of the scene, distinguishing the water environment from the surrounding area [87]. It is possible to propose individual detectors tailored for different motion states. Despite various limitations, this method can still successfully locate and track swimmers in each frame. The proposed approach remains effective at locating and tracking swimmers in every frame [88]. Furthermore, one integration approach applied a Raspberry Pi using a USB camera for acquiring input images. This method performs image recognition for activity identification through deep learning, and a buzzer is utilized for sounding alarms [89].

In the context of neural network learning, for instance, a lightweight convolutional encoder for drowning detection was proposed to achieve unsupervised monitoring in unmanned swimming pools. Due to the urgency of the rescue process in an unmanned pool when a drowning event occurs and the potential lack of immediate assistance in unattended pools, the system utilizes a collection of time series videos acquired from underwater cameras. The results indicated that this method achieved good overall performance [90]. To effectively monitor swimming pools at night, a method was developed to accurately detect people in infrared television images. This approach involved creating three different datasets: two from infrared televisions placed on public beaches and one from forward-looking infrared (FLIR) cameras located on pedestrian bridges. To achieve accurate results, a pixel-level classifier based on a CNN was used for precise person detection [91]. In the study, five convolutional neural networks, namely, SqueezeNet [92], GoogLeNet [93], AlexNet [94], ShuffleNet [95] and ResNet50 [96], were used. These networks were trained and executed based on the collected dataset. Among these models, ResNet50 demonstrated the best performance, achieving 100 % prediction accuracy within a reasonable amount of training time [97]. Additionally, four machine learning models were compared in terms of their ability to predict nonfatal drowning risks. These models included logistic regression (10.13039/501100009319LR) [98], the random forest (RF) [99], the support vector machine (SVM) and stack-based models. The stacking ensemble model outperformed the other three base models on the nonfatal drowning dataset [100]. The study proposed a swimmer detection method that utilizes local motion and intensity information estimated from an image sequence. Local motion information is obtained through the computation of dense optical flows and cyclic graphs. A heuristic approach is employed to generate motion maps representing local motion, including ripples or image pixel movements over short periods (random/static) [101]. A swimmer detection method that combines mean-shift clustering and cascaded reinforcement learning algorithms was proposed and validated using image sequences recorded in an actual indoor swimming pool [102].

In terms of image processing, computer vision can detect the activity status of a drowning person in real time in an emergency situation [103], and in drowning scenarios, common challenges include foreground detection and high noise in behavior recognition. Therefore, for aquatic backgrounds or crowded pool scenes, a series of methods, such as background subtraction, denoising, data fusion, and blob segmentation, have been proposed [104]. To handle noise, a method based on robust estimation was proposed, leveraging the high stability of robust estimation against noise. By combining robust estimation techniques with image restoration methods, an adaptive robust image smoothing algorithm was introduced. Experimental results indicated that this algorithm can smooth the original image while effectively preserving the original image data [105]. Eliminating water surface reflections can also increase the accuracy of drowning detection systems. One approach involves establishing a more refined water ripple background model, while another method entails enhancing the water ripple background [106]. By using the Bayesian framework and a high-order Markov model with a mixed transition distribution, one can improve drowning detection systems. By leveraging this model, it becomes possible to partially or fully recover objects hidden in mirror reflections while suppressing the foreground errors caused by dynamic backgrounds. This yields enhanced foreground detection, and experimental results have demonstrated that this technique achieves excellent performance both during the day and at night [107]. For situations in which noise backgrounds frequently appear on the water surface, a new segmentation method was proposed. The key feature of this segmentation method is based on processing the hue components of colors in the hue/saturation/value (HSV) color space in predefined image regions. This method is not affected by water surface reflections, splashes, random water flow movements, or overall scene lighting changes [108].

In larger outdoor water environments, the integration of mature drone technology has become feasible. Real-time drowning recognition models and algorithms can be employed using drones, making them more suitable for outdoor locations such as beaches, especially when compared to current drowning models [109]. Larger bathing beaches can use ocean monitoring technology and automatic drowning detection methods; they can employ artificial neural networks to train and test networks for detecting drowning swimmers [110]. During nighttime in unattended areas, it is feasible to monitor daytime swimmers' activities and send an alert if someone unintentionally falls into the water [111].

On another note, data can be acquired based on images, such as by using the scalable MapReduce programming model. The MapReduce model parameterizes the interior search algorithm (ISA) by distributing a dataset into equally disjoint sets. Another method for attaining improved speed is the use of interest point detectors to extract "important" features from a video [112]. Moreover, research has also utilized the Microsoft Common Objects in Context (MS COCO) dataset as a starting point for training a transfer learning method. A novel object detector for outdoor swimmers was developed using this approach. The model was then evaluated and retrained to classify people into categories such as swimmers, potential swimmers, and pedestrians [113]. Additionally, the features of swimmers can be extracted and evaluated based on criteria such as the normal swimming speeds assessed from swimmer time series, an method not restricted by camera angles for evaluating an upright position, and rules for assessing dangerous situations. These methods can effectively detect swimmers in distress at an early stage [114].

**Fig. 4:**
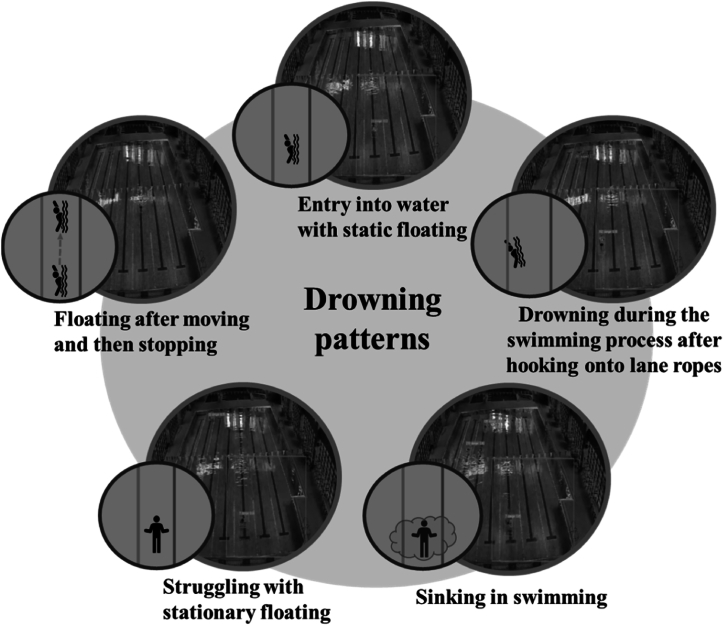
Computer vision is a field that involves using images for various forms of AI applications, such as object tracking, image recognition, and neural network training. One application of computer vision involves drowning prevention. With advancements in technology, the speed of image recognition has greatly improved. Regarding system implementation, the Insight Vision team utilized image recognition methods to achieve a drowning prevention and early warning system. Based on the practical experience of experts and site managers, the Insight Vision team categorized drowning patterns into multiple types, as illustrated in the diagram. The five main drowning patterns depicted include entry into water with static floating, floating after moving and then stopping, drowning during the swimming process after hooking onto lane ropes, struggling with stationary floating and sinking, and remaining still at the bottom of the water during the swimming process. All these patterns are based on results obtained from on-site field tests. Through such a computer vision system, early drowning situation detection is possible, which can provide timely alerts to enhance the monitoring and management of pool safety.

Such a system analyzes various drowning patterns, while even experienced lifeguards may not fully understand the postures of drowning people. The system relies on the practical experience of experts and field managers to analyze drowning patterns and establish a warning system. The system, referencing the practical experience of field managers, identifies five drowning patterns, which cover 90 % of drowning situations. As shown in Fig. 4, the five drowning patterns are as follows. Type 1 involves entry into the water while still floating, type 2 is movement followed by stopping and floating, type 3 is drowning after becoming entangled in lane ropes during swimming, type 4 involves struggling to float or sink in place, and type 5 is submersion to the bottom while swimming and remaining still. All of these patterns are based on real-world field test results.

## 4 Real-time drowning alerts achieved through the IoT-based integration of the internet and device connectivity

**Fig. 5:**
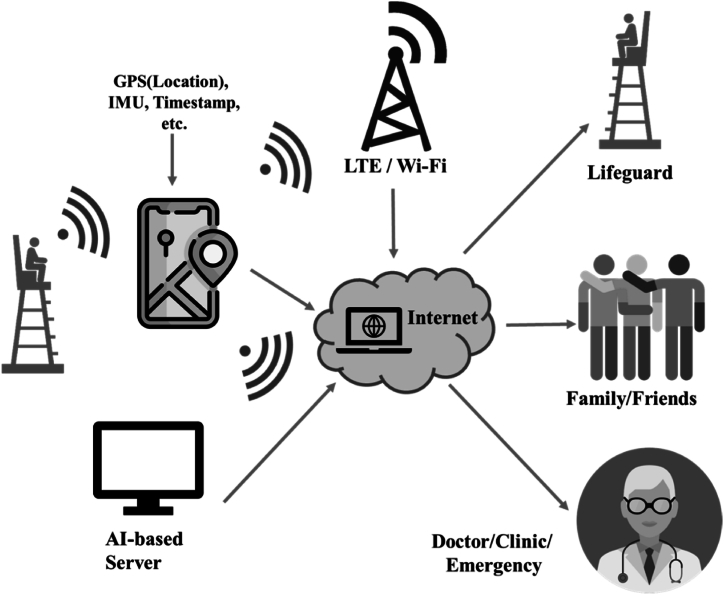
Illustration of an IoT application concept. With the widespread application of 5G technology, the IoT field is undergoing significant transformation. The high-speed transmission of 5G signals indeed brings tremendous convenience to the IoT. With a transmission speed that is approximately 10 times faster than that of 4G, 5G facilitates more convenient applications in the IoT, allowing for the positions of wearable devices or relevant physiological signals to be linked to the network. This enables family members, doctors, or friends to receive real-time information, ensuring that alarms can be promptly received. These instant communication and data sharing capabilities are crucial for emergency rescue operations, contributing to reductions in the probability of fatalities, such as in drowning incidents. Furthermore, the 5G communication network supports a larger number of simultaneous connected devices, which is highly beneficial for large-scale IoT applications. For instance, in scenarios where a significant number of people are engaged in activities near the water, multiple wearable devices can simultaneously connect to the network. This allows relevant personnel to promptly receive real-time information, further enabling them to monitor and report potential hazardous situations.

With the increasingly fast network speeds available in today's world, alerts can be rapidly transmitted upon the detection of a drowning incident in a pool, enabling lifeguards or other personnel to be promptly notified. Research has also demonstrated the widespread applications of the IoT, as shown in Fig. 5.

In the realm of the IoT, the IoT for drowning swimmers refers to the advancement and utilization of the IoT for creating drowning prevention devices and solutions. This field centers around facilitating machine-to-machine communication, harnessing big data, and employing machine learning techniques to enhance the efficiency and reliability of control centers. By leveraging this technology, we can gather more precise information pertaining to air quality, water quality, potential risks, and climate changes, thus enabling proactive measures for preventing drowning incidents. In these IoT applications, devices are typically deployed over wider geographic areas, and they may also possess mobility capabilities [115]. A proposed student tracking and monitoring system for schools utilizes radio-frequency identification (RFID) technology. This system incorporates IoT technology and cloud computing, enabling families, schools, and authorities to easily monitor students' attendance and track their movements in real-time, ensuring their safety [116]. A cost-effective system utilizing wireless sensors and actuators was devised to oversee and regulate the quality of swimming pools. This innovative system aims to minimize the need for extensive staff involvement in pool maintenance tasks. Moreover, an Android mobile application was developed, granting users the ability to remotely monitor and manage swimming pool conditions in real time. This facilitates prompt data analysis, enabling the establishment of specific thresholds for each parameter. Users are promptly notified when these thresholds are surpassed [117]. Another study proposed a type of autonomous portable IoT-based device that can monitor people engaging in water activities in real time. In the event of danger, it sends alerts to the responsible team, thereby preventing potentially fatal consequences during activities [118]. The camera is also integrated with underwater robotic vehicle technology. The underwater vehicle utilizes an external camera system to detect drowning incidents on the water surface and then dispatches the robot to the location at which an event occurs [119]. In a drowning prevention system, the IoT and transfer learning can be utilized to provide a continuous pool monitoring system. Models pretrained on ImageNet [120] can be employed to achieve higher accuracy, sensitivity, and precision [121]. In another study concerning automated drowning prevention systems, a small number of sensors were utilized in conjunction with Arduino. These sensors can monitor the pH value of a pool area in real time. By monitoring the pH value, swimmers can safely engage in activities, and under the IoT framework, the system can observe more environmental states [122]. In today's era of high-speed communication, with speeds reaching 5G, systems can leverage combinations of 5G and deep learning models. An instant drowning notification system can detect and classify situations where parents or caregivers are not paying attention, reminding them to actively supervise their children in a pool. The proposed model could successfully classify seven patterns with very high accuracy [123].

The system designed by SK Yaswanthkumar and others based on application platforms utilizes two main concepts for automatically detecting drowning incidents. One is sonar sound navigation and ranging detection, and the other is thermal detection. Both of these detection technologies are employed for underwater human detection [124]. In contrast, a swimmer's condition can also be monitored through physiological signals. Heart rate variability (HRV) is especially useful for gaining insight into both the physiological and psychological states of swimmers. By examining heart rate variations, we can obtain reliable information about the functioning of the autonomic nervous system (ANS). HRV provides a convenient measure for understanding an individual's overall physiological status [125]. The Poseidon drowning detection system in the United States is the world's first computer system designed to help prevent drowning incidents in public swimming pools. The system's scene reconstruction accuracy was evaluated using both synthetic and real-world scenarios, and it demonstrated high 3D positioning and reconstruction precision [126]. Quantitative descriptions were also used to model intermediate semantic behaviors.

A. H. Kam utilized a novel regression-based approach with a modified version of a functional connectivity network to demonstrate sophisticated behavioral reasoning. Compared to other competitive decision-making solutions, this method showed the ability to rapidly learn and accurately classify information [127]. In terms of pool-related equipment, the framework comprises an array of proximity sensors in an elevator housing, a laser gate line module, a pull switch on the pool sidewall (used to detect anyone on the bottom surface of the pool), a drainage motor, an alarm light combination, and a buzzer. During the nonoperational hours of the pool, the elevator component is kept at the top of the swimming pool to prevent children or pets from accidently falling into the pool [128].

## 5 Current global drowning prevention systems

**Fig. 6:**
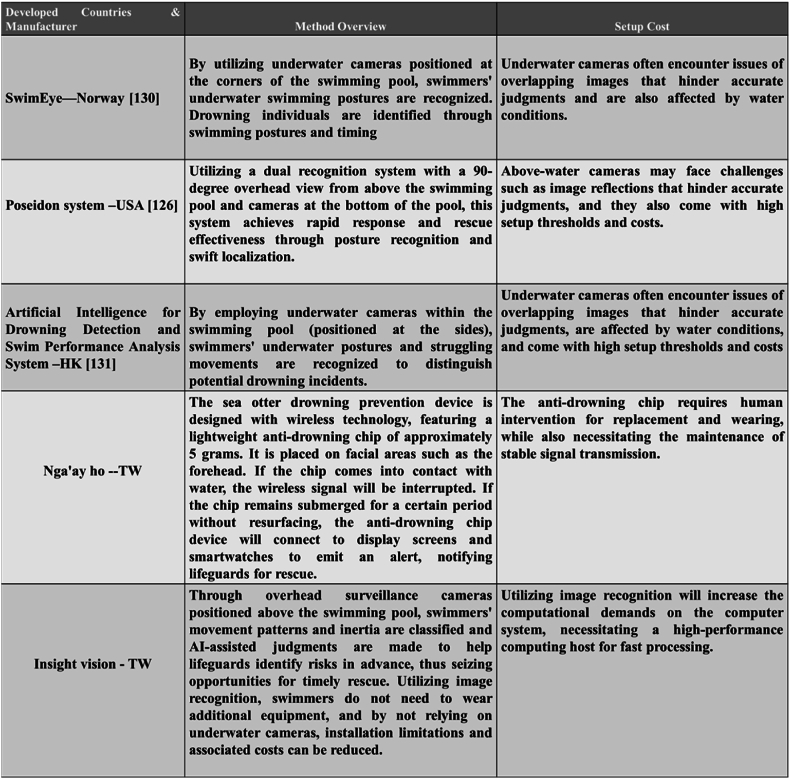
Drowning prevention systems are now in practical use worldwide. Among the current global drowning prevention systems, the SwimEye drowning prevention system in Norway [129] transmits images taken by underwater cameras to a computer system that uses the OpenPose algorithm to recognize the human body. The system monitors the postures and movements of bathers to detect any potentially dangerous situations. When the system determines a hazard, an early warning status is triggered, and lifeguards are notified if necessary. The Poseidon system in the United States [126] attaches a camera to the wall of a pool to capture pictures of the deep water area, while another camera is installed in a higher position to monitor the shallow water area to ensure full coverage of the pool. All cameras operate synchronously to accurately monitor the situation inside the pool. If someone stands still at the bottom of the pool for 10 s, the system automatically triggers an alarm. The Hong Kong Artificial Intelligence Drowning Detection and Swimming Performance Analysis System [130] uses images acquired from underwater cameras to track swimmers' postures, allowing them to be rescued from drowning when they are in danger underwater. Considering that the compatible methods in the field mostly use image recognition-related applications, most swimming images also come from images captured by underwater cameras, but the cost of underwater cameras is higher than that of other cameras, and it is not easy to identify when bathers' activity areas overlap. Insight Vision Co., Ltd proposed a drowning prevention system that can capture the activity statuses and related position parameters of actual bathers by setting the camera frame at a high level, which can save more costs than an underwater camera. By setting up a camera at a tall height, the system can clearly identify the various activity postures of swimmers, calculate the swimmers' movement according to the customized drowning algorithm, and issue an alarm so that lifeguards can rescue a drowning person in time.

Fig. 6 illustrates the use of underwater cameras in drowning prevention systems. Currently, systems in Norway and at Hong Kong University use underwater cameras for drowning prevention. The SwimEye system in Norway transmits images captured by underwater cameras to a computer system and then employs the OpenPose algorithm for human pose recognition. The system monitors the poses and movements of swimmers to detect potential dangers. When the system identifies a danger, it triggers an alert and notifies lifeguards if necessary. The system uses OpenPose for recognizing body poses and detecting struggling movements based on swimmers' pose activities, thereby determining if a swimmer is in danger at the bottom of the pool. When a swimmer is in danger, a yellow alert state is triggered, and after a brief countdown, the drowning alert turns red and is sent directly to the pool lifeguards for an immediate rescue.

However, the high cost of installing underwater cameras and the overlap of swimmers' activity areas pose challenges. In Hong Kong, an artificial intelligence-based drowning detection and swimming performance analysis system also uses underwater cameras to recognize swimmers' underwater activity patterns. The system extracts clear pose information for swimmers and calculates their safety levels based on the amounts of time that swimmers spend underwater. A longer underwater time raises the alert level, notifying lifeguards to pay attention. Similar to the SwimEye system, it faces challenges when swimmers overlap in the same area. The Poseidon drowning prevention system in the United States installs cameras both above the pool and on the pool floor, providing a dual recognition system with a 90-degree overhead view. This system achieves rapid responses and rescues through posture recognition and quick positioning. The cameras installed on the pool walls capture deep water images, while the overhead cameras monitor shallower scenes, providing a complete picture. If someone stays still at the bottom of the pool for 10 s, an alarm is triggered. However, the challenges faced include the feasibility of installing cameras with 90-degree overhead views and the high cost of system installation.

In Taiwan, the Sea Otter drowning prevention device is a wireless technology design. When a chip is immersed in water, its wireless signal is blocked. If a person does not resurface within a certain amount of time, the drowning prevention chip device connects to the display screen and smartwatch to send an alarm, notifying lifeguards to perform a rescue. However, the manual replacement and wearing of drowning prevention chips, as well as the stability of signal transmission, present challenges. As drowning prevention systems integrate AI technology due to a shortage of lifeguard personnel, the goal is to utilize the computational power and stability of AI technology to achieve enhanced efficiency and speed. Significant advancements in computer vision have addressed the previous graphics processing shortcomings. Drowning events form a globally recognized issue, making drowning prevention a priority for every country.

In addition, the AI-based drowning prevention system developed by Insight Vision Co., Ltd. in Taiwan aims to classify swimmer movements and inertial patterns via overhead monitoring cameras, assisting lifeguards in identifying risks early and seizing rescue opportunities. The need for additional equipment is eliminated by the system's use of image recognition algorithms. Additionally, the use of monitoring cameras instead of underwater cameras reduces installation costs and restrictions. The system establishes a drowning algorithm based on a swimmer's position and speed, effectively calculating their current speed and position to determine whether the swimmer is safely active. In the event of a drowning swimmer, the system triggers a network transmission to the alert system, which activates warning lights and sounds to alert the relevant pool staff, aiding lifeguards in observing each swimmer in the area to prevent drowning events.

## 6 Summary

Technological advances have led to improvements in anti-drowning systems, which previously required users to wear specific equipment in order to make timely measurements. Now, however, embedded systems and computer-vision-based artificial intelligence have flourished, making swimmers more comfortable during activities without the need to wear equipment. For the development of this technology, cost control must be considered. The cost of sensors in embedded systems is relatively low, but the cost of sensor wear and replacement equipment needs to be considered. Artificial intelligence applications using computer vision can achieve instant anti-drowning detection through image recognition, which requires a certain level of development costs and almost no need to replace equipment, providing another feasible solution. However, when a swimming pool is crowded and has many users, it is necessary to maintain real-time identification and tracking to ensure real-time alerts and prevent drowning. Some methods of using sensors may slow down interpretation or even delay message transmission due to the large amount of received data that needs to be analyzed. Computer vision can solve this problem, but for dense occlusion situations, the algorithm may need to be strengthened to maintain accuracy. In addition, if it can be combined with Internet of Things applications, an unmanned swimming pool monitoring system can be constructed, thereby reducing reliance on lifeguards, improving the speed of responses to drowning incidents, and better preventing drowning.

The anti-drowning systems designed by the institutes reviewed in this article are mainly experimented with and applied to closed stadium swimming pools and similar environments. Although these studies have achieved certain results in experiments in closed venues, in open areas without clear boundaries, such as beaches, bathing areas, etc., the monitoring scope and use conditions of these studies have been limited in practical applications. First, monitoring in open areas faces greater challenges because the scale and instability of the area may make it difficult to install and operate monitoring facilities, which in turn will affect the operational efficiency and accuracy of the system. Secondly, water characteristics and environmental conditions in open sites are more diverse than in closed sites, and factors such as weather, tides, currents, and water quality limit the applicability of existing anti-drowning systems in these sites. These are also areas where researchers can continue to improve and engage in research and development.

Future research on anti-drowning systems in open waters can draw on the results and experience of previous research. Intelligent sensing technologies such as visual sensing, sonar and radar can be combined with embedded devices and set up on water surface monitoring towers or buoys to perform water monitoring. These sensors can be integrated with embedded systems to process and analyze the monitored data through the embedded devices and then issue alarms or notify supervisors. Additionally, combining deep learning techniques with water simulations can improve model accuracy and predictive capabilities. By training the neural network to learn the characteristics and patterns of water flow; model the flow speed, flow direction, tide and other data in the water area; and conduct simulation and analysis of the water area based on the prediction results of the neural network, the problems of the environment and boundaries can be addressed, thereby enabling more accurate predictions and judgments [131]. Drowning prevention is a global issue. In the future, research and design methods can be examined in a variety of different fields and combined to improve the effectiveness of drowning prevention.

## CRediT authorship contribution statement

**Wei-Chun Kao:** Writing – review & editing, Writing – original draft, Formal analysis, Data curation. **Yi-Ling Fan:** Writing – review & editing, Writing – original draft, Validation, Data curation, Formal analysis, Investigation, Supervision. **Fang-Rong Hsu:** Writing – original draft, Supervision. **Chien-Yu Shen:** Writing – review & editing, Writing – original draft, Supervision, Conceptualization. **Lun-De Liao:** Writing – review & editing, Writing – original draft, Validation, Supervision, Resources, Project administration, Methodology, Funding acquisition, Conceptualization.

## Declaration of competing interest

The authors declare the following financial interests/personal relationships which may be considered as potential competing interests: Lun-De Liao reports financial support and administrative support were provided by 10.13039/501100004737National Health Research Institutes, Taiwan. Lun-De Liao reports a relationship with National Health Research Institutes that includes: employment. If there are other authors, they declare that they have no known competing financial interests or personal relationships that could have appeared to influence the work reported in this paper.
